# On the Weak Convergence of the Extragradient Method for Solving Pseudo-Monotone Variational Inequalities

**DOI:** 10.1007/s10957-017-1214-0

**Published:** 2018-01-18

**Authors:** Phan Tu Vuong

**Affiliations:** Institute of Statistics and Mathematical Methods in Economics, Vienna University of Technology, Wiedner Hauptstraße 8, 1040 Vienna, Austria

**Keywords:** Variational inequality, Extragradient method, Pseudo-monotonicity, Weak convergence, 47J20, 49J40, 49M30

## Abstract

In infinite-dimensional Hilbert spaces, we prove that the iterative sequence generated by the extragradient method for solving pseudo-monotone variational inequalities converges weakly to a solution. A class of pseudo-monotone variational inequalities is considered to illustrate the convergent behavior. The result obtained in this note extends some recent results in the literature; especially, it gives a positive answer to a question raised in Khanh (Acta Math Vietnam 41:251–263, 2016).

## Introduction

Variational inequalities serve as a powerful mathematical model, which unifies important concepts in applied mathematics like systems of nonlinear equations, necessary optimality conditions for optimization problems, complementarity problems, obstacle problems, or network equilibrium problems [1]. Therefore, this model has numerous applications in the fields of engineering, mathematical programming, network economics, transportation research, game theory, and regional sciences [2].

Several techniques for the solution of a variational inequality (VI) in finite-dimensional spaces have been suggested such as projection method, extragradient method, Tikhonov regularization method and proximal point method; see, e.g., [1]. Typically, for guaranteeing the convergence to a solution of the VI, some kinds of monotonicity of the assigned mapping is required. In case of gradient maps, generalized monotonicity characterizes generalized convexity of the underlying function [3]. The well-known gradient projection method can be successfully applied for solving strongly monotone VIs and inverse strongly monotone VIs [1, 4]. In practice, these assumptions are rather strong. The Tikhonov regularization and proximal point methods can serve as an efficient solution method for solving monotone VIs. For pseudo-monotone VIs, however, it may happen that every regularized problem generated by the Tikhonov regularization (resp. every problem generated by the proximal point method) is not pseudo-monotone [5]. This implies that the regularization procedures performed in Tikhonov regularization and proximal point methods may destroy completely the given pseudo-monotone structure of the original problem and can make auxiliary problems more difficult to solve than the original one.

To overcome this drawback, Korpelevich introduced the extragradient method [6]. In the original paper, this method was applied for solving monotone VIs in finite-dimensional spaces. It is a known fact [1, Theorem 12.2.11] that the extragradient method can be successfully applied for solving pseudo-monotone VIs. Because of its importance, extragradient-type methods have been widely studied and generalized [1].

Recently, the extragradient method has been considered for solving VIs in infinite-dimensional Hilbert spaces [7–9]. Providing that the VI has solutions and the assigned mapping is monotone and Lipschitz continuous, it is proved that the iterative sequence generated by the extragradient method converges weakly to a solution. However, as stated in [9, Section 6, Q2], it is not clear if the weak convergence is still available when monotonicity is replaced by pseudo-monotonicity. The aim of this paper is to give a positive answer to this question. As a consequence, the scope of the related optimization problems can be enlarged from convex optimization problems to pseudo-convex optimization problems. This guarantees the advantage of extragradient method in comparing with the other solution methods.

The paper is organized as follows: We first recall some basic definitions and results in Sect. 2. The weak convergence of the extragradient method for solving pseudo-monotone, Lipschitz continuous VIs is discussed in Sect. 3. An example is presented in Sect. 4 to illustrate the behavior of the extragradient method. We conclude the note with some final remarks in Sect. 5.

## Preliminaries

Let *H* be real Hilbert space with inner product $\langle \cdot , \cdot \rangle$ and induced norm $\Vert .\Vert$ and *K* be a nonempty, closed and convex subset of *H*. For each $u\in H$, there exists a unique point in *K* (see [2, p. 8]), denoted by $P_K(u)$, such that

$$\begin{aligned} \Vert u-P_K(u)\Vert \le \Vert u-v\Vert \quad \forall v\in K. \end{aligned}$$

It is well known [2, 10] that the projection operator can be characterized by

1 $$\begin{aligned} \langle u-P_K(u),v-P_K(u) \rangle \le 0 \quad \forall v\in K. \end{aligned}$$

Let $F: H \rightarrow H$ be a mapping. The variational inequality VI(*K*, *F*) defined by *K* and *F* consists in finding a point $u^*\in K$ such that

2 $$\begin{aligned} \langle F(u^*), u-u^*\rangle \ge 0 \quad \forall u\in K. \end{aligned}$$

The solution set of VI(*K*, *F*) is abbreviated to Sol(*K*, *F*).

### Remark 2.1

$u^*\in \mathrm{Sol}(K, F)$ if and only if $u^*=P_K(u^*-\lambda F(u^*))$ for all $\lambda >0$.

We recall some concepts which are useful in the sequel.

### Definition 2.1

The mapping $F: H \rightarrow H$ is said to be

- pseudo-monotone if
  $$\begin{aligned} \langle F(u),v-u\rangle \ge 0\;\Rightarrow \; \langle F(v),v-u\rangle \ge 0 \quad \forall u,v \in H; \end{aligned}$$
- monotone if
  $$\begin{aligned} \langle F(u)-F(v), u-v\rangle \ge 0\quad \forall u,v \in H; \end{aligned}$$
- Lipschitz continuous if there exists $L>0$ such that
  $$\begin{aligned} \Vert F(u)-F(v)\Vert \le L \Vert u-v \Vert \quad \forall u,v \in H; \end{aligned}$$
- sequentially weakly continuous if for each sequence $\{u^n\}$ we have: $\{u^n\}$ converges weakly to *u* implies $\{F(u^n)\}$ converges weakly to *F*(*u*).

### Remark 2.2

It is clear that monotonicity implies pseudo-monotonicity. However, the converse does not hold. For example, the mapping $F:\left]0,+\infty \right[ \rightarrow \left]0,+\infty \right[$, defined by $F(u)=\frac{a}{a+u}$ with $a>0$ is pseudo-monotone but not monotone.

We recall a result which is called Minty lemma [11, Lemma 2.1].

### Proposition 2.1

Consider the problem VI(*K*, *F*) with *K* being a nonempty, closed, convex subset of a real Hilbert space *H* and $F: K\rightarrow H$ being pseudo-monotone and continuous. Then, $u^*$ is a solution of VI(*K*, *F*) if and only if

$$\begin{aligned} \langle F(u), u-u^*\rangle \ge 0 \quad \forall u\in K. \end{aligned}$$

## Weak Convergence of the Extragradient Method

In this section, we consider the problem VI(*K*, *F*) with *K* being nonempty, closed, convex and *F* being pseudo-monotone on *H* and Lipschitz continuous with modulus $L>0$ on *K*. We also assume that the solution set Sol(*K*, *F*) is nonempty.

**Extragradient Algorithm**

- **Data:** $u^0\in K$ and $\{ \lambda _k \} \in [a,b]$, where $0<a\le b<1/L$.
- **Step 0:** Set $$k=0$$.
- **Step 1:** If $u^k=P_K(u^k-\lambda _k F(u^k))$ then stop.
- **Step 2:** Otherwise, set
  $$\begin{aligned} \bar{u}^k= & {} P_K(u^k-\lambda _k F(u^k)),\\ u^{k+1}= & {} P_K(u^k-\lambda _k F(\bar{u}^k)). \end{aligned}$$

Replace *k* by $k+1$; go to **Step 1**.

### Remark 3.1

If at some iteration we have $F(u^k)=0$, then $u^k=P_K(u^k-\lambda _k F(u^k))$ and the Extragradient Algorithm terminates at step *k* with a solution $u^k$. From now on, we assume that $F(u^k)\not =0$ for all *k* and the Extragradient Algorithm generates an infinite sequence.

We recall an important property of the iterative sequence $\{u^k\}$ generated by the Extragradient Algorithm; see, e.g., [6, 9].

### Proposition 3.1

Assume that *F* is pseudo-monotone and *L*-Lipschitz continuous on *K* and Sol(*K*, *F*) is nonempty. Let $u^*$ be a solution of VI(*K*, *F*). Then, for every $k\in \mathbb {N}$, we have

3 $$\begin{aligned} \Vert u^{k+1}-u^*\Vert ^2\le \Vert u^k-u^*\Vert ^2-\left( 1-\lambda _k^2L^2\right) \Vert u^k-\bar{u}^{k}\Vert ^2. \end{aligned}$$

We are now in the position to establish the main result of this note. The following theorem states that the sequence $\{u^k\}$ converges weakly to a solution of VI(*K*, *F*). This result extends the Extragradient Algorithm for solving monotone VIs [7, 9] to pseudo-monotone VIs.

### Theorem 3.1

Assume that *F* is pseudo-monotone on H, sequentially weakly continuous and *L*-Lipschitz continuous on *K*. Assume also that Sol(*K*, *F*) is nonempty. Then, the sequence $\{u^k\}$ generated by the Extragradient Algorithm converges weakly to a solution of VI(*K*, *F*).

### Proof

Since $0<a \le \lambda _k \le b <1/L$, it holds that

$$\begin{aligned} 0<1-b^2L^2 \le 1-\lambda _k^2L^2 \le 1-a^2L^2 <1. \end{aligned}$$

Therefore, from Proposition 3.1, the sequence $\{u^k\}$ is bounded and

$$\begin{aligned} \lim _{k \rightarrow \infty }\Vert u^k-\bar{u}^{k}\Vert =0. \end{aligned}$$

Since *F* is Lipschitz continuous on *K* we have

$$\begin{aligned} \Vert F(u^k)-F(\bar{u}^{k})\Vert \le L \Vert u^k-\bar{u}^{k}\Vert . \end{aligned}$$

Hence

$$\begin{aligned} \lim _{k \rightarrow \infty } \Vert F(u^k)-F(\bar{u}^{k})\Vert =0. \end{aligned}$$

As $\{u^k\}$ is a bounded sequence in a Hilbert space, there exists a subsequence $\{u^{k_i}\}$ of $\{u^k\}$ converging weakly to an element $\hat{u} \in K$. Since $\lim _{k \rightarrow \infty }\Vert u^k-\bar{u}^{k}\Vert =0$, $\{\bar{u}^{k_i}\}$ also converges weakly to $\hat{u}$. We will prove that $\hat{u} \in \mathrm{Sol}(K,F)$. Indeed, since

$$\begin{aligned} \bar{u}^k=P_K(u^k-\lambda _k F(u^k)), \end{aligned}$$

by the projection characterization (1), it holds

$$\begin{aligned} \left\langle u^{k_i}-\lambda _{k_i} F(u^{k_i})-\bar{u}^{k_i}, v-\bar{u}^{k_i} \right\rangle \le 0 \quad \forall v \in K, \end{aligned}$$

or equivalently,

$$\begin{aligned} \frac{1}{\lambda _{k_i}}\left\langle u^{k_i}-\bar{u}^{k_i}, v-\bar{u}^{k_i} \right\rangle \le \left\langle F(u^{k_i}),v-\bar{u}^{k_i} \right\rangle \quad \forall v \in K. \end{aligned}$$

This implies that

4 $$\begin{aligned} \frac{1}{\lambda _{k_i}}\left\langle u^{k_i}-\bar{u}^{k_i}, v-\bar{u}^{k_i} \right\rangle +\left\langle F(u^{k_i}),\bar{u}^{k_i}-u^{k_i} \right\rangle \le \left\langle F(u^{k_i}),v-u^{k_i} \right\rangle \quad \forall v \in K. \end{aligned}$$

Fixing $v \in K$ and letting $i \rightarrow +\infty$ in the last inequality, remembering that $\lim _{k \rightarrow \infty }\Vert u^k-\bar{u}^{k}\Vert =0$ and $\lambda _k \in [a,b] \subset ]0,1/L[$ for all *k*, we have

5 $$\begin{aligned} \liminf _{i \rightarrow \infty } \left\langle F(u^{k_i}),v-u^{k_i} \right\rangle \ge 0. \end{aligned}$$

Now we choose a sequence $\{\epsilon _i\}_i$ of positive numbers decreasing and tending to 0. For each $\epsilon _i$, we denote by $n_i$ the smallest positive integer such that

6 $$\begin{aligned} \left\langle F(u^{k_j}),v-u^{k_j} \right\rangle + \epsilon _i \ge 0 \quad \forall j \ge n_i, \end{aligned}$$

where the existence of $n_i$ follows from (5). Since $\left\{ \epsilon _i \right\}$ is decreasing, it is easy to see that the sequence $\left\{ n_i \right\}$ is increasing. Furthermore, for each *i*, $F(u^{k_{n_i}}) \not = 0$ and, setting

$$\begin{aligned} v^{k_{n_i}}=\frac{F(u^{k_{n_i}})}{\Vert F(u^{k_{n_i}})\Vert ^2}, \end{aligned}$$

we have $\left\langle F(u^{k_{n_i}}),v^{k_{n_i}} \right\rangle =1$ for each *i*. Now we can deduce from (6) that for each *i*

$$\begin{aligned} \left\langle F(u^{k_{n_i}}),v+\epsilon _i v^{k_{n_i}} -u^{k_{n_i}} \right\rangle \ge 0, \end{aligned}$$

and, since *F* is pseudo-monotone, that

7 $$\begin{aligned} \left\langle F(v+\epsilon _iv^{k_{n_i}}),v+\epsilon _i v^{k_{n_i}} -u^{k_{n_i}} \right\rangle \ge 0. \end{aligned}$$

On the other hand, we have that $\left\{ u^{k_i} \right\}$ converges weakly to $\hat{u}$ when $i \rightarrow \infty$. Since *F* is sequentially weakly continuous on *K*, $\left\{ F(u^{k_i}) \right\}$ converges weakly to $F(\hat{u})$. We can suppose that $F(\hat{u}) \not = 0$ (otherwise, $\hat{u}$ is a solution). Since the norm mapping is sequentially weakly lower semicontinuous, we have

$$\begin{aligned} \Vert F(\hat{u}) \Vert \le \lim \inf _{i \rightarrow \infty } \Vert F(u^{k_i})\Vert . \end{aligned}$$

Since $\left\{ u^{k_{n_i}} \right\} \subset \left\{ u^{k_i} \right\}$ and $\epsilon _i \rightarrow 0$ as $i \rightarrow 0$, we obtain

$$\begin{aligned} 0 \le \lim _{i \rightarrow \infty } \Vert \epsilon _i v^{k_{n_i}}\Vert = \lim _{i \rightarrow \infty } \frac{\epsilon _i}{\Vert F(u^{k_{n_i}})\Vert } \le \frac{0}{\Vert F(\hat{u}) \Vert }=0. \end{aligned}$$

Hence, taking the limit as $i \rightarrow \infty$ in (7), we obtain

$$\begin{aligned} \left\langle F(v),v-\hat{u} \right\rangle \ge 0. \end{aligned}$$

It follows from Proposition 2.1 that $\hat{u} \in \mathrm{Sol}(K,F)$.

Finally, we prove that the sequence $\{u^k\}$ converges weakly to $\hat{u}$. To do this, it is sufficient to show that $\{u^k\}$ cannot have two distinct weak sequential cluster points in Sol(*K*, *F*). Let $\{u^{k_j}\}$ be another subsequence of $\{u^k\}$ converging weakly to $\bar{u}$. We have to prove that $\hat{u}=\bar{u}$. As it has been proven above, $\bar{u} \in \mathrm{Sol}(K,F)$. From Proposition 3.1, the sequences $\{\Vert u^k-\hat{u}\Vert \}$ and $\{\Vert u^k-\bar{u}\Vert \}$ are monotonically decreasing and therefore converge. Since for all $k \in \mathbb {N}$,

$$\begin{aligned} 2\left\langle u^{k},\bar{u}-\hat{u} \right\rangle = \Vert u^k-\hat{u}\Vert ^2- \Vert u^k-\bar{u}\Vert ^2+\Vert \bar{u}\Vert ^2-\Vert \hat{u}\Vert ^2, \end{aligned}$$

we deduce that the sequence $\{\left\langle u^{k},\bar{u}-\hat{u} \right\rangle \}$ also converges. Setting

$$\begin{aligned} l=\lim _{k \rightarrow \infty } \left\langle u^{k},\bar{u}-\hat{u} \right\rangle , \end{aligned}$$

and passing to the limit along $\{u^{k_i}\}$ and $\{u^{k_j}\}$ yields, respectively,

$$\begin{aligned} l= \left\langle \hat{u},\bar{u}-\hat{u} \right\rangle =\left\langle \bar{u},\bar{u}-\hat{u} \right\rangle . \end{aligned}$$

This implies that $\Vert \hat{u}-\bar{u}\Vert ^2=0$ and therefore $\hat{u}=\bar{u}$.$\square$

### Remark 3.2

The author in [13] studied the extragradient method for solving strongly pseudo-monotone variational inequalities with the following choice of stepsizes:

$$\begin{aligned} \sum _{k=0}^{\infty } \lambda _k=\infty , \quad \lim _{k \rightarrow \infty } \lambda _k=0. \end{aligned}$$

It was proved that the iterative sequence generated by the extragradient method converges strongly to a solution. By considering an example [13, Example 4.2], the author stated that the condition $\lim _{k \rightarrow \infty } \lambda _k=0$ cannot be omitted. We have shown that if this condition is violated then the strong convergence reduces to the weak convergence.

It is also worth stressing that, the basic extragradient method can serve as an adequate solution method for solving pseudo-monotone VIs, which was not guaranteed by the method studied in [13].

### Remark 3.3

If we replace the Lipschitz continuity of *F* on *K* by its Lipschitz continuity on the whole space *H*, then the conclusion in Theorem 3.1 still holds for the subgradient extragradient method [7]. Indeed, a careful reviewing shows that Lemma 5.2 in [7] is also guaranteed for pseudo-monotone mappings instead of monotone ones (see also [12]). The conclusion can be obtained by using a similar technique as in Theorem 3.1.

### Remark 3.4

When the function *F* is monotone, it is not necessary to impose the sequential weak continuity on *F*. Indeed, in that case, it follows from (4) and the monotonicity of *F* that

$$\begin{aligned} \frac{1}{\lambda _{k_i}}\left\langle u^{k_i}-\bar{u}^{k_i}, v-\bar{u}^{k_i} \right\rangle +\left\langle F(u^{k_i}),\bar{u}^{k_i}-u^{k_i} \right\rangle\le & {} \left\langle F(u^{k_i}),v-u^{k_i} \right\rangle \\\le & {} \left\langle F(v),v-u^{k_i} \right\rangle \quad \forall v \in K. \end{aligned}$$

Letting $i \rightarrow +\infty$ in the last inequality, remembering that $\lim _{k \rightarrow \infty }\Vert u^k-\bar{u}^{k}\Vert =0$ and $\lambda _k \in [a,b] \subset ]0,1/L[$ for all *k*, we have

$$\begin{aligned} \left\langle F(v),v-\hat{u} \right\rangle \ge 0\quad \forall v \in K. \end{aligned}$$

## An Illustrative Example

In this section, we present an example to illustrate the main results obtained in Sect. 3. Another example can be found in [9, Example 5.2], where the mapping *F* is monotone and Lipchitz continuous. The following example is considered in [13], where the mapping *F* is pseudo-monotone but not monotone.

Let $H=\ell _2$, the real Hilbert space, whose elements are the square-summable sequences of real numbers, i.e., $H=\{u=(u_1,u_2,\ldots ,u_i,\ldots ): \sum _{i=1}^{\infty }|u_i|^2<+\infty \}$. The inner product and the norm on *H* are given by setting

$$\begin{aligned} \langle u,v\rangle =\sum _{i=1}^{\infty }u_iv_i \quad \text { and } \quad \Vert u\Vert =\sqrt{\langle u, u\rangle } \end{aligned}$$

for any $$u=(u_1,u_2,\ldots ,u_i,\ldots ), v=(v_1,v_2,\ldots ,v_i,\ldots )\in H$$.

Let $\alpha ,\beta \in \mathbb {R}$ be such that $\displaystyle \beta>\alpha>\frac{\beta }{2}>0$. Put

$$\begin{aligned} K_\alpha =\{u\in H: \Vert u\Vert \le \alpha \}, \quad F_\beta (u)=(\beta -\Vert u\Vert )\,u, \end{aligned}$$

where $\alpha$ and $\beta$ are parameters. It is easy to verify that $F_\beta$ is sequentially weakly continuous on *H* and $\mathrm{Sol}(K_\alpha ,F_\beta )=\{0\}.$ Note that $F_\beta$ is Lipschitz continuous and pseudo-monotone on $K_\alpha$. Indeed, for any $u, v\in K_\alpha$,

$$\begin{aligned} \Vert F_\beta (u)-F_\beta (v)\Vert= & {} \Vert (\beta -\Vert u\Vert )u-(\beta -\Vert v\Vert )v\Vert \\= & {} \Vert \beta (u-v)-\Vert u\Vert (u-v)-(\Vert u\Vert -\Vert v\Vert )v\Vert \\\le & {} \beta \Vert u-v\Vert +\Vert u\Vert \Vert u-v\Vert +|\Vert u\Vert -\Vert v\Vert |\Vert v\Vert \\\le & {} \beta \Vert u-v\Vert +\alpha \Vert u-v\Vert +\Vert u-v\Vert \alpha \\= & {} (\beta +2\alpha )\Vert u-v\Vert . \end{aligned}$$

Hence, $F_\beta$ is Lipschitz continuous on $K_\alpha$ with the Lipschitz constant $L:=\beta +2\alpha$. Let $u,v\in K_\alpha$ be such that $\langle F_\beta (u), v-u\rangle \ge 0$. Then

$$\begin{aligned} (\beta -\Vert u\Vert ) \langle u, v-u\rangle \ge 0. \end{aligned}$$

Since $\Vert u\Vert \le \alpha <\beta$, we have $\langle u, v-u\rangle \ge 0$. Hence,

$$\begin{aligned} \langle F_\beta (v), v-u\rangle= & {} (\beta -\Vert v\Vert )\langle v, v-u\rangle \\\ge & {} (\beta -\Vert v\Vert )(\langle v, v-u\rangle -\langle u, v-u\rangle )\\\ge & {} (\beta -\alpha )\Vert u-v\Vert ^2 \ge 0. \end{aligned}$$

We have thus shown that $F_\beta$ is pseudo-monotone on $K_\alpha$. It is worthy to stress that $F_\beta$ is not monotone on $K_\alpha$. To see this, it suffices to choose $u=(\frac{\beta }{2}, 0, \ldots , 0, \ldots ), v=(\alpha ,0, \ldots ,0, \ldots )\in K_\alpha$ and note that

$$\begin{aligned} \langle F_\beta (u)-F_\beta (v), u-v\rangle =\left( \frac{\beta }{2}-\alpha \right) ^3<0. \end{aligned}$$

We now apply the Extragradient Algorithm for solving the variational inequality VI($K_{\alpha }, F_{\beta }$). We choose $\lambda _k=\lambda \in \left] 0,\frac{1}{L}\right[=\left] 0,\frac{1}{\beta +2\alpha }\right[$ and we take starting point as any $u^0 \in K_{\alpha }$. The projection onto $K_{\alpha }$ is explicitly calculated as

8 $$\begin{aligned} P_{K_\alpha }u= \left\{ \begin{array}{ll} u, &{}\quad \text {if} \quad \Vert u\Vert \le \alpha ,\\ \frac{\alpha u}{\Vert u\Vert }, &{}\quad \text {otherwise.} \end{array}\right. \end{aligned}$$

Since for all *k*,

$$\begin{aligned} 0<\lambda<\frac{1}{\beta +2\alpha }<\frac{1}{\beta -\Vert u^k\Vert }, \end{aligned}$$

then we have

$$\begin{aligned} \Vert u^k-\lambda F_{\beta }(u^k)\Vert =\left( 1-\lambda \left( \beta -\Vert u^k\Vert \right) \right) \Vert u^k \Vert \le \Vert u^k \Vert \le \alpha . \end{aligned}$$

Therefore,

$$\begin{aligned} \bar{u}^k=P_{K_\alpha }(u^k-\lambda _k F(u^k))=\left( 1-\lambda \left( \beta -\Vert u^k\Vert \right) \right) u^k. \end{aligned}$$

Similarly, we can deduce that

$$\begin{aligned} \Vert u^k-\lambda _k F_ \beta (\bar{u}^k)\Vert \le \alpha . \end{aligned}$$

Indeed, we have

$$\begin{aligned} u^k-\lambda _k F_ \beta (\bar{u}^k)=u^k-\lambda \left( \beta -\Vert \bar{u}^k\Vert \right) \left( 1-\lambda \left( \beta -\Vert u^k\Vert \right) \right) u^k. \end{aligned}$$

Since

9 $$\begin{aligned} 1-\lambda \left( \beta -\Vert \bar{u}^k\Vert \right) \left( 1-\lambda \left( \beta -\Vert u^k\Vert \right) \right)= & {} 1-\lambda \left( \beta -\Vert \bar{u}^k\Vert \right) \nonumber \\&+\,\lambda ^2\left( \beta -\Vert \bar{u}^k\Vert \right) \left( \beta -\Vert u^k\Vert \right) \nonumber \\\ge & {} 1-\lambda \left( \beta -\Vert \bar{u}^k\Vert \right) >0, \end{aligned}$$

we can write

$$\begin{aligned} \Vert u^k-\lambda _k F_ \beta (\bar{u}^k)\Vert =\left[ 1-\lambda \left( \beta -\Vert \bar{u}^k\Vert \right) \left( 1-\lambda \left( \beta -\Vert u^k\Vert \right) \right) \right] \Vert u^k\Vert \le \Vert u^k\Vert \le \alpha . \end{aligned}$$

This and (9) imply that

10 $$\begin{aligned} \nonumber \Vert u^{k+1}\Vert= & {} \Vert P_{K_\alpha }(u^k-\lambda _k F_ \beta (\bar{u}^k))\Vert \\ \nonumber= & {} \Vert u^k-\lambda \left( \beta -\Vert \bar{u}^k\Vert \right) \bar{u}^k \Vert \\= & {} \left[ 1- \lambda \left( \beta -\Vert \bar{u}^k\Vert \right) \left( 1-\lambda \left( \beta -\Vert u^k\Vert \right) \right) \right] \Vert u^{k}\Vert . \end{aligned}$$

We have

11 $$\begin{aligned} \lambda \left( \beta -\Vert \bar{u}^k\Vert \right) \left( 1-\lambda \left( \beta -\Vert u^k\Vert \right) \right) \nonumber= & {} \lambda \left( \beta -\Vert \bar{u}^k\Vert \right) \left( 1-\lambda \beta +\lambda \Vert u^k\Vert \right) \\\ge & {} \lambda \left( \beta -\Vert \bar{u}^k\Vert \right) \left( 1-\lambda \beta \right) \\ \nonumber= & {} \lambda \left( \beta -(1-\lambda \beta )\Vert u^k\Vert -\lambda \Vert u^k\Vert ^2 \right) \left( 1-\lambda \beta \right) . \end{aligned}$$

Considering the function $f(x):=\beta -\left( 1-\lambda \beta \right) x-\lambda x^2$ with $x\in [0,\alpha ]$, it is easy to see that *f* is decreasing on $[0,\alpha ]$. Therefore, the minimal value of *f* is

$$\begin{aligned} \beta -\left( 1-\lambda \beta \right) \alpha -\lambda \alpha ^2, \end{aligned}$$

which is attained at $x=\alpha$. Combining this with (11) and (10) yields

12 $$\begin{aligned} \nonumber \Vert u^{k+1}\Vert\le & {} \left( 1-\lambda \left( \beta -\left( 1-\lambda \beta \right) \alpha -\lambda \alpha ^2 \right) \left( 1-\lambda \beta \right) \right) \Vert u^k\Vert \\ \nonumber= & {} \left( 1- \left( \lambda \beta - \lambda \alpha + \lambda ^2 \alpha \beta - \lambda ^2 \alpha ^2 \right) \left( 1-\lambda \beta \right) \right) \Vert u^k\Vert \\= & {} \left[ 1-\left( \beta -\alpha \right) \lambda \left( 1+\alpha \lambda \right) \left( 1-\lambda \beta \right) \right] \Vert u^k\Vert . \end{aligned}$$

We claim that

$$\begin{aligned} q:=\left( \beta -\alpha \right) \lambda \left( 1+\alpha \lambda \right) \left( 1-\lambda \beta \right) \in ]0,1[. \end{aligned}$$

Indeed, since $\alpha <\beta$ and $0<\lambda <\frac{1}{\beta +2\alpha }$, we have $q>0$. To verify that $q<1$, it is sufficient to show that $\left( \beta -\alpha \right) \lambda \left( 1+\alpha \lambda \right) <1$. Since $\beta /2< \alpha <\beta$ and $0<\lambda <\frac{1}{\beta +2\alpha }$ we have

$$\begin{aligned} \left( \beta -\alpha \right) \lambda \left( 1+\alpha \lambda \right)< & {} \left( \beta -\alpha \right) \frac{1}{\beta +2\alpha }\left( 1+\frac{\alpha }{\beta +2\alpha }\right) \\< & {} \frac{\beta }{2}\frac{1}{\beta +\beta }\left( 1+\frac{\beta }{\beta +\beta }\right) =\frac{3}{8}. \end{aligned}$$

This implies that $q \in ]0,1[$ and we can deduce from (12) that

$$\begin{aligned} \Vert u^k\Vert \le \left( 1-q \right) ^k \Vert u^0\Vert , \end{aligned}$$

for all $k\in \mathbb {N}$. This means that the sequence $\{u^k\}$ converges strongly to 0, the unique solution of VI($K_{\alpha }, F_{\beta }$).

## Conclusions

We have considered the extragradient method for solving infinite-dimensional variational inequalities with a pseudo-monotone and Lipschitz continuous mapping. We have shown that the iterative sequence generated by the extragradient method converges weakly to a solution of the considered variational inequality, provided that such a solution exists. The strong convergence of the iteration sequence is still an open question that could be an interesting topic for a future research.
